# Cognitive-behavioral phenotypes of Williams syndrome are associated with genetic variation in the GTF2I gene, in a healthy population

**DOI:** 10.1186/s12868-014-0127-1

**Published:** 2014-11-28

**Authors:** Bernard J Crespi, Peter L Hurd

**Affiliations:** Department of Biology, Simon Fraser University, 8888 University Drive, Burnaby, V5A 1S6, BC Canada; Department of Psychology, and Neuroscience and Mental Health Institute, University of Alberta, 116 St. and 85 Avenue, Edmonton, T6G 2R3 AB Canada

**Keywords:** Social behavior, Anxiety, Williams syndrome, Autism, GTF2I gene

## Abstract

**Background:**

Individuals with Williams syndrome, a neurogenetic condition caused by deletion of a set of genes at chromosomal location 7q11.23, exhibit a remarkable suite of traits including hypersociality with high, nonselective friendliness and low social anxiety, expressive language relatively well-developed but under-developed social-communication skills overall, and reduced visual-spatial abilities. Deletions and duplications of the Williams-syndrome region have also been associated with autism, and with schizophrenia, two disorders centrally involving social cognition. Several lines of evidence have linked the gene GTF2I (*General Transcription Factor IIi*) with the social phenotypes of Williams syndrome, but a role for this gene in sociality within healthy populations has yet to be investigated.

**Results:**

We genotyped a large set of healthy individuals for two single-nucleotide polymorphisms in the GTF2I gene that have recently been significantly associated with autism, and thus apparently exhibit functional effects on autism-related social phenotypes. GTF2I genotypes for these SNPs showed highly significant association with low social anxiety combined with reduced social-communication abilities, which represents a metric of the Williams-syndrome cognitive profile as described from previous studies.

**Conclusions:**

These findings implicate the GTF2I gene in the neurogenetic basis of social communication and social anxiety, both in Williams syndrome and among individuals in healthy populations.

## Background

A promising approach to elucidating the genetic bases of human social behavior is the study of well-defined genetic or genomic variation with specific cognitive, behavioral and neurological effects. Williams syndrome represents a paradigmatic neurogenetic condition for such analyses, as it is caused by hemizygous deletion (loss of one copy, from the normal diploid complement of two copies) of about 25 genes at 7q11.23, and its social cognitive-behavioral profile has been thoroughly characterized as involving hypersociality and low social anxiety with high, nonselective friendliness, expressive language relatively well-developed but social-communication skills reduced overall, high levels of non-social anxiety, and notably-reduced visual-spatial abilities [1-7]. In contrast to these effects of gene deletion in Williams syndrome, duplication of the same set of genes has been associated with a directly-contrasting cognitive-behavioral profile of selectively-underdeveloped expressive language and high levels of separation anxiety [8-10]. These findings suggest the presence of dosage-sensitive genes in the Williams-syndrome region that affect anxiety, language and social behavior in two opposite directions, in individuals with this syndrome as well as in healthy populations. Imaging studies of individuals with Williams syndrome provide evidence that these cognitive and behavioral phenotypes are mediated by abnormalities in the insula, orbitofrontal cortex and amygdala, with reduced amygdala activation to threatening faces, abnormal orbitofrontal modulation of social inhibition and engagement, and altered structure and function in regions of the insula that are correlated with measures of the Williams syndrome personality profile [11-13].

Genes mediating cognitive-behavioral phenotypes in 7q11.23 deletions and duplications have been identified in several ways. First, the cognitive-behavioral profiles of individuals with small, atypical deletions in this region have implicated the GTF2I gene in hypersociality [14,15]. Variable-sized deletions have also been generated in mouse models, which have implicated the region including GTF2I, GTF2IRD1, LIMK1, and intervening genes, in sociality and anxiety phenotypes [16].

Second, mouse single-gene knockouts have indicated that GTF2I gene dosages modulate levels of sociality, with hemizygosity leading to higher levels of less-discriminate social interaction [17], and triploidy, which corresponds to the duplication, being associated with higher social anxiety [9].

Third, studies of segregating genetic variation for genes within the Williams-syndrome deletion region have identified an association of several SNPs in GTF2I with autism [18]. These results are of notable interest given reports of autism spectrum behavior in individuals with Williams syndrome [19-21], and evidence for association of duplications for this 7q11.23 region with autism in case–control studies of (predominantly) children [22]. Duplications of this 7q11.23 region have also recently been associated with increased risk of schizophrenia (in adults) [23]. Links of Williams-syndrome region genes and CNVs with autism and schizophrenia suggest that the phenotypes constituting these disorders may be relevant to those of individuals with 7q11.23 deletions and duplications.

Taken together, these lines of evidence regarding the causes of the Williams syndrome region cognitive-behavioral phenotypes suggest important roles for the GTF2I gene. One approach to uncovering these roles is to evaluate the cognitive-behavioral effects of polymorphisms in the GTF2I gene in healthy populations, which have yet to be investigated. In this study, we have tested for association of the two autism-associated SNPs in the GTF2I gene [18] with a set of autism-associated and schizophrenia-associated psychological phenotypes as defined by and derived from subscales of the Autism Quotient (AQ) [24] and the Schizotypal Personality Questionnaire - Brief Revised (SPQ) [25].

From the SPQ, we analyzed variation in the Social Anxiety subscale, which quantifies a core phenotype associated with the Williams-syndrome region (low versus high social anxiety). From the AQ, we analyzed the Communication and Social subscales, based on the well-documented social-interactive phenotypes of Williams syndrome, and the previously-established association of these two SNPs with autism [18]. We predicted that these previously-established autism-associated alleles and genotypes of GTF2I [18] should be associated with higher scores for these two measures.

We also constructed a metric of Social Anxiety relative to Communication, based on a conceptual model of Williams syndrome developed by Reilly et al. [26,27] and Fishman et al. [28], which posits that language and conversation skills, and drive for social approach and engagement, are jointly fundamental to the Williams syndrome cognitive-behavioral phenotypes. We constructed this metric by scaling the Communication subscale, and the Social Anxiety subscale, each to between 0 and 1 through dividing by the highest value of each (to equalize their ranges and relative contributions to the score). We then reversed the Social Anxiety score (by subtracting it from 1), and added this modified Social Anxiety score to the Communication score. For this variable, relatively high values thus represent relatively low social anxiety combined with reduced adaptive social-communicative abilities, which can be regarded as the cognitive-behavioral combination most-closely characteristic of Williams syndrome, from previous studies [6,7,29]. This metric is referred to here as Williams syndrome profile score, and we predicted that higher scores should be associated with the autism-associated alleles and genotypes [18]. Finally, we analyzed GTF2I genotypes in relation to total Autism score from the AQ, and total Schizotypy score from the SPQ, based on the associations of copy number variants in this region with autism and schizophrenia [22,23], and the studies linking Williams syndrome with some autistic traits as discussed above. For these metrics, we predicted that total Autism scores should be higher for individuals with the autism-associated alleles or genotypes [18], and that the alternative alleles or genotypes should be associated with total Schizotypy. This prediction is predicated on a model whereby the GTF2I genotypes analyzed here are associated with alterations to GTF2I gene dosage or activity in some way (as evidenced by [18]), in a manner that is comparable, but considerably less pronounced, to the opposite alterations observed in CNV deletions and duplications. Comparable subclinical effects of genetic variants have been described previously for CNVs mediating risk of autism and schizophrenia [30].

## Methods

### Populations and sampling

Questionnaire data and saliva samples used for DNA extraction were collected from Caucasian undergraduate students at the University of Alberta and Simon Fraser University (311 females and 177 males). All protocols were carried out according to guidelines established by ethics boards of the both universities (Simon Fraser University Office of Research Ethics, Permit 2010 s0554, and University of Alberta Arts, Science and Law Research Ethics Board, Permit Pro00015728), and all participants provided informed consent. Given the low frequencies of Williams syndrome region deletions (1 in about 10,000-20,000 births [2,4]) and duplications (1 in over 20,000 births) [23], and the fact that both CNVs engender penetrant phenotypes that are readily ascertained in early childhood, it is exceedingly unlikely that anyone in our sample population harbored either of these CNVs.

### Questionnaire data and analyses

Schizotypy was measured using the Schizotypal Personality Questionnaire-Brief Revised (SPQ-BR), autistic phenotypes were measured using the Autism Spectrum Quotient (AQ). From the AQ, we analyzed the Social skills and Communication subscales, in accordance with the social and communication dimensions of Williams syndrome phenotypes.

### SNP genotype data and analyses

Genomic DNA was extracted from mouthwash samples that were provided by each participant, and stored at -20C. Genotyping was conducted on two SNPs in GTF2I (rs4717907 and rs13227433). These two GTF2I SNPs were chosen for genotyping because their common alleles were significantly associated with autism by Malenfant et al. [18]. Genotyping was performed by Genome Québec (Montréal, Canada) using the Sequenom Mass-ARRAY iPlex platform. The two GTF2I SNPs were in high linkage disequilibrium (r^2^ = 0.94), which is consistent with LD results from SNAP Pairwise LD analysis of 1000 genomes CEU data (Broad Institute 2008, http://www.broadinstitute.org/mpg/snap/ldsearchpw.php). Genotype data were analyzed using ANOVA and under two alternative models of gene action with regard to allelic dominance. Haplotype-based analysis using hapassoc [31] yielded essentially equivalent results to analyses using each marker separately. Statistical analyses were performed using R, version 2.15.1. Both markers were in Hardy-Weinberg equilibrium (*χ*^2^ = 0.73 for rs4717907, 0.55 for rs13227433, P >0.05 for both).

## Results

The means and standard deviations for each of the variables analyzed are presented in Table 1, and ANOVA significance values under alternative genetic models of dominance, codominance and recessiveness are provided in Table 2. Under a codominant model, genotypes of the GTF2I single nucleotide polymorphisms rs4717907 and rs13227433 were not significantly different by ANOVA for the Social, Communication, or Social Anxiety subscale scores, or for total Autism or total Schizotypy, although the differences for Communication (for rs13227433) and for Social Anxiety (for both SNPs) approached significance (0.05 < P <0.10) (Table 1). By contrast, Williams syndrome profile scores (measuring high AQ-Communication in combination with low SPQ-Social Anxiety) were highly significantly different between genotype groups under the codominant model (P = 0.0053 for rs4717907 and P = 0.0069 for rs13227433) (Table 1). Individuals with the common (autism-associated) alleles and genotypes thus showed higher scores on this profile, which indicates lower Social Anxiety combined with higher Communication scores (i. e., reduced communication abilities).

**Table 1:** **Means and standard deviations (in parentheses) for the different SNP genotypes of GTF2I**

	**Genotypes**					
	**rs4717907**			**rs13227433**		
**Questionnaire metrics (ranges)**	**CC**	**CT**	**TT**	**AA**	**AC**	**CC**
AQ-Social (0–9)	2.62 (1.77)	2.34 (1.63)	2.51 (1.98)	2.60 (1.76)	2.29 (1.57)	2.63 (2.03)
AQ-Communication (0–9)	2.41 (1.93)	2.13 (1.76)	1.83 (1.84)	2.40 (1.90)	2.06 (1.75)	1.89 (1.90)
SPQ-Social Anxiety (4–20)	10.94 (3.75)	10.65 (3.62)	12.17 (4.25)	10.84 (3.77)	10.56 (3.61)	12.03 (4.21)
Williams syndrome profile score (0.1–1.36)	0.721 (0.217)	0.704 (0.206)	0.595 (0.234)	0.725 (0.215)	0.701 (0.203)	0.609 (0.235)
AQ-Total (4–36)	17.60 (5.47)	16.90 (5.42)	17.06 (6.15)	17.56 (5.43)	16.53 (5.44)	17.37 (6.25)
SPQ-Total (34–125)	82.03 (15.08)	79.97 (16.49)	82.76 (15.53)	81.76 (14.99)	80.36 (16.03)	81.51 (16.49)

**Table 2:** **P-values from ANOVAs comparing mean values of psychological variables between genotypes**

	**Genetic marker, model**					
	**rs4717907**			**rs13227433**		
**Psychological variable**	**CODOM**	**DOM**	**REC**	**CODOM**	**DOM**	**REC**
AQ-Social	0.28	0.98	0.13	0.14	0.57	0.10
AQ-Communication	0.12	0.15	0.06	0.08	0.24	0.03*
SPQ-Social Anxiety	0.09	0.04*	0.94	0.09	0.04*	0.91
Williams syndrome profile score	0.0053**	0.0017**	0.08	0.0069**	0.0033*	0.04*
AQ-Total	0.44	0.79	0.20	0.14	0.80	0.08
SPQ-Total	0.36	0.58	0.29	0.64	0.90	0.39

CODOM refers to codominant model (ANOVA with 2 df), DOM and REC refer to models that pool heterozygotes with one of the homozygotes (1 df).

Boldface highlights statistical significance, with * for P < 0.05 and ** for P < 0.01.

Variation in GTF2I genotypes for rs4717907 and rs13227433 was nominally associated with Social Anxiety under a dominant model (P = 0.04 for each), and rs13227433 genotype was nominally associated with Communication under a recessive model (P = 0.03) (Tables 1 and 2). Under a dominant model, there were highly significant differences between genotypes for the Williams syndrome profile score (Tables 1 and 2); under a recessive model, rs13227433 showed nominal significance for this metric, and rs4717907 showed marginal non-significance. There were no associations between GTF2I genotypes and the other variables under either model, although the association of rs13227433 with total Autism approached significance (P = 0.08) under a recessive model.

## Discussion

Our results demonstrate significant associations of Williams syndrome cognitive-behavioral phenotypes with autism-associated SNP polymorphisms in the GTF2I gene. In particular, especially under codominant and dominant models, GTF2I genotypes were strongly associated with the Williams syndrome profile metric of low Social Anxiety scores (which are indicative of relatively high social motivation) combined with high Communication scores (which are indicative of relatively ‘autistic’ phenotypes as regards conversational interactions). These results are thus consistent with the hypothesis that high social approach motivation (and concomitant lower levels of fear and anxiety in social contexts), coupled with reduced social-interactive abilities, represent a central aspect of Williams syndrome cognitive-behavioral phenotypes [26-28], and specifically implicate genetic variation in the GTF2I gene in this phenotype. In this context, our results from a healthy population are compatible with multiple independent lines of evidence on the role of GTF2I in mediating social phenotypes of Williams syndrome, including data from both mouse models and genotype-phenotype correlations of individuals with atypical deletions, as described in more detail above. Our data thus also implicate GTF2I in the neurological basis of social behavior in Williams syndrome, based on documented alterations to activation and integration of the insula, orbitofrontal cortex and amygdala in this syndrome [11-13], although this hypothesis requires targeted study using imaging genetics of individuals who differ in genotype for rs4717907 and rs13227433.

Our findings also provide independent support, in a healthy population, for the demonstration by Malefant et al. [18] that the SNPs rs4717907 and rs13227433 show functional associations with phenotypes that are highly relevant to Williams syndrome, and extend these results by showing association with a more-specific phenotype that was predicted from previous studies [26-28] to characterize behavior in this syndrome. As such, these results provide convergent evidence for behavioral-genetic continuity between the effects of these GTF2I polymorphisms in Williams syndrome, autism, and healthy populations, and indicate that functional characterization of the effects of these SNPs should provide insights into the genetic bases of variation in human social cognition and behavior, in both healthy and clinical populations. An intriguing aspect of the phenotypes mediated by these polymorphisms is that they involve a combination of higher propensity to engage in social interactions (and lower social anxiety) with reduced social abilities, a manifestation of pleiotropy that may appear counterintuitive but reflects behavioral phenotypes observed in both Williams syndrome and in a well-established subgroup within autism referred to as ‘active-but-odd’ (e.g., [32]). Our findings thus suggest that healthy populations exhibit comparable, genetically-based, behavioral variation, though in highly-attenuated form.

Limitations of our study include a lack of validation of our Williams syndrome cognitive phenotype index among individuals with Williams syndrome, although data based on self-report in this context may be problematic to obtain and interpret. In addition, larger sample sizes, and testing in additional non-clinical populations (in addition to the autism populations analyzed by Malenfant et al. [18]) would be useful for more-robust interpretation.

The functional bases for cognitive-behavioral effects from GTF2I remains largely unknown. GTF2I codes for the transcription factor TFII-I that is highly expressed in the brain [33], and it belongs to a small paralogous gene family of transcriptional regulators that includes two other genes in the Williams-syndrome region, GTF2IRD1 and GTF2IRD2 [4] that interact with one another in their effects [15]. Of particular interest with regard to the cognitive-behavioral effects of GTF2I is the finding that this gene shows evidence of genomic imprinting effects, with higher expression from the maternally-inherited allele [34]. Williams-syndrome region deletions are thus expected to render gene-expression levels relatively paternally-biased (compared to typically-developing individuals), which may be related to the high levels of social approach and solicitation, coupled with underdeveloped social abilities, in Williams syndrome [35]. Genomic and gene expression studies have also identified a series of changes to GTF2I family genes along the human lineage [4], as well as a striking increase in gene expression of GTF2I in humans compared to related primates [36,37]. These findings, considered together with our results, imply that the GTF2I gene is also involved in the evolution of complex behavior among humans, in part through its effects on personality traits related to social approach, social anxiety, and social cognition more generally.

## Conclusions

The present study provides evidence that polymorphisms in the GTF2I gene mediate social anxiety in relation to social abilities in healthy populations, which, together with convergent results from previous studies, implicates this gene in the hypersocial cognitive-behavioral phenotype of Williams syndrome.
